# Overlap between ultra-processed food and food that is high in fat, salt or sugar: analysis of 11 annual waves of the UK National Diet and Nutrition Survey 2008/2009–2018/2019

**DOI:** 10.1136/bmjnph-2024-001035

**Published:** 2025-03-28

**Authors:** Viktorija Kesaite, Yanaina Chavez-Ugalde, Martin White, Jean Adams

**Affiliations:** 1 MRC Epidemiology Unit, University of Cambridge, Cambridge, UK; 2 MRC Epidemiology Unit, University of Cambridge School of Clinical Medicine, Cambridge, UK; 3 Institute of Health & Society, Newcastle University, Newcastle upon Tyne, UK; 4 University of Cambridge School of Clinical Medicine, Cambridge, UK

**Keywords:** Nutrition assessment, Nutrient deficiencies, Malnutrition

## Abstract

While many countries use guidance and policies based on nutrients and food groups to support citizens to consume healthy diets, fewer have explicitly adopted the concept of ultra-processed foods (UPF). UPF consumption is associated with many adverse health outcomes in cohort studies. In the UK, a nutrient profiling model (NPM) is used to identify foods high in fat, salt or sugar (HFSS) and several policies target these. It is not known how well the NPM also captures UPF. We aimed to quantify the proportion of food and drink items consumed in the UK that are HFSS, UPF, both or neither and describe the food groups making the largest contributions to each category. We analysed data from the National Diet and Nutrition Survey, between 2008/2009 and 2018/2019, using descriptive statistics. We used three metrics of food consumption: all foods, percentage of energy in all foods (reflecting that different foods are consumed in different portion sizes and are of different energy densities) and percentage of food weight in all foods (reflecting that some UPFs have few calories but are consumed in large volumes). We found that 33.4% of foods, 47.4% of energy and 16.0% of food weight were HFSS; 36.2%, 59.8% and 32.9%, respectively, were UPFs; 20.1%, 35.1% and 12.6% were both and 50.5%, 27.9% and 63.7% were neither. In total, 55.6% of UPF foods, 58.7% of energy from UPFs and 38.3% of food weight from UPF consumed were also HFSS. The most common food groups contributing to foods that were UPF but not HFSS were low-calorie soft drinks and white bread. The UK NPM captures at best just over half of UPFs consumed in the UK. Expanding the NPM to include ingredients common in UPFs (eg, non-nutritive sweeteners, emulsifiers) would capture a larger percentage of UPFs and could incentivise ‘deformulation’ of UPF products.

**WHAT IS ALREADY KNOWN ON THIS TOPIC**

- In many high-income countries, including the UK, consumption of ultra-processed food (UPF) and foods high in fat, salt or sugar (HFSS) constitute more than half of all calories consumed.
- Despite emerging evidence of the negative health impacts of UPF, current UK diet and obesity policy focuses on reducing consumption of HFSS foods.

**WHAT THIS STUDY ADDS**

- To our knowledge, this is the first study to examine the extent to which the UK nutrient profiling model (NPM) also identifies UPFs. We found that the NPM captures over half of UPFs based on all foods consumed and percent of energy, but only about a third based on food weight. Among all foods consumed, the most common food groups that were UPF but not HFSS were low-calorie soft drinks and white bread. Other types of bread (eg, brown, wholemeal) and high-fibre breakfast cereals were also common.

**HOW THIS STUDY MIGHT AFFECT RESEARCH, PRACTICE OR POLICY**

- Further consideration and potential inclusion of those UPFs that are not under the scope of the current definition of HFSS (eg, foods containing non-nutritive sweeteners, emulsifiers) could enable existing policies to have a wider reach.

## Introduction

 Numerous studies have shown that diets high in some components (eg, sodium, sugar, fat and calories) and low in others (eg, whole grains and fruits) increase the risk of non-communicable diseases such as cardiovascular disease and cancer.^1^ Recent estimates suggest that ultra-processed foods (UPFs) and drinks (hereafter: ‘foods’) constitute more than half of total energy intake among individuals in the UK.^2^ Similar estimates are observed in the consumption of foods that are high in fat, salt or sugar (HFSS).^3^

In the UK, a nutrient profiling model (NPM) is used to identify HFSS foods and restrict advertising on television,^4^ out-of-doors in some local authorities and where foods can be placed in grocery stores.^5^ The NPM was originally developed in 2004/2005 and defines foods as being HFSS based on energy, saturated fat, total sugar, sodium, fibre, protein, and fruit, vegetable, and nut content per 100 g.^6^ An updated version was developed in 2018,^7^ based on revised guidance on sugar and fibre intake.^8^ However, despite consultations, this has not yet been adopted into practice.^9^

High consumption of UPF, as identified by the Nova classification,^10^ has also been negatively associated with adverse health outcomes.^11^ The Nova system is the most common method of classifying foods based on their degree of food processing used in the academic literature and has been identified as the most applicable to the UK.^9^ Recent Euromonitor statistics suggest that the highest volume of sales of UPFs globally are in Western Europe, North America and Australasia, with increasing trends observed elsewhere.^12^ Findings based on a systematic review indicate that the USA and the UK had the highest proportion of energy intake from UPFs (>50%).^13^

The UK’s approach to public health nutrition, where nutrients and food groups such as fruit and vegetables are used to drive guidance and policy reflects similar approaches in many countries. However, a growing number of countries have now also included specific reference to avoidance of UPFs in their dietary guidance.^14^

As policy-makers contend with whether to focus policy and dietary guidance on UPFs, an important question is: how well do current approaches, such as the UK’s NPM, also identify UPFs? If most UPFs are also HFSS then the benefits of introducing additional UPF-focused policy and guidance may be minimal. We are aware of only one other study examining the overlap between UPFs and other methods of identifying less-healthy foods.^15^ This recent US study found that around three-quarters of UPF foods purchased were also HFSS and that extending the HFSS definition to include non-nutritive sweeteners, flavourings, colours and additives would capture nearly 100% of UPFs.^15^ To the best of our knowledge, no previous work has explored the overlap between HFSS, as identified by the UK NMP and UPF in the UK.

In this study, we have two aims: (1) to quantify the proportion of foods consumed in the UK that are HFSS, UPF, both or neither and (2) to assess the percentage of per capita daily foods derived from HFSS, UPF, both or neither, and how this varies for males and females and across age groups.

## Methods

We used data from eleven waves (2008/2009–2018/2019) of the UK National Diet and Nutrition Survey (NDNS).

### Data description

NDNS is a continuous cross-sectional survey conducted every year in the UK. It collects data on food consumption, nutrient intake and nutritional status of the general population aged 1.5 years and over residing in private UK households. Each year, a multistage probability design is used to generate a new random sample. Selected household addresses are clustered into small geographical units called primary sampling units. Households are randomly selected from these units, and participants are randomly selected from each household. Full data are collected from about 500 children and 500 adults each year. All participants aged 16 or above in the NDNS survey provided informed consent before answering any survey questions. For children between the ages of 4 and 15, consent was obtained both from the child and their parent or guardian, and for toddlers between the ages of 1.5 and 3, consent was obtained from their parents or guardians.^16^

In 2008–2019, all NDNS participants self-reported their food and beverage consumption over 3 or four days using a written food diary, with portion sizes recorded using standard household measures and product labels. Parents and guardians reported on behalf of children under 11 years. Food diary data are entered into the Diets In Nutrients Out database and food codes are used to link foods to the NDNS nutrient composition database, which includes nutritional information on more than 6000 foods.^17^ Participants also provided some information about whether food was homemade, fresh, frozen or ready-made. The response rate to food diaries is 50% or more. We used study weights provided in the NDNS dataset (which adjust for non-selection and non-response). In the subgroup analyses, we recalculated weights following the guidance in the NDNS study weights guide documentation.^17 18^

### Categorisation of foods as HFSS and UPF

We used the UK NPM to classify foods as HFSS or not. The NPM was originally developed by the Food Standards Agency in 2004/2005 to provide the Office of Communications (Ofcom) with guidance and recommendations on the composition of foods that could be advertised to children in the UK.^6^ It has since been used more widely to define HFSS foods for a range of UK policy measures.^19^ The HFSS classification is an objective algorithm based on nutrient content; calculation of HFSS status using the NPM 2004/2005 involves three steps: (1) calculation of ‘A’ points (from energy, saturated fat, total sugar and sodium content); (2) calculation of ‘C’ points (from fruit, vegetables and nuts; fibre and protein content) and (3) subtraction of ‘C’ points from ‘A’ points. Foods that have a score of 4 or greater, and beverages that have a score of 1 or greater are classified as HFSS or ‘less healthy’; other foods are classified as not HFSS or ‘heathier’.^6^ We also used an updated version of the NPM 2018 which includes free sugars rather than total sugars, salt instead of sodium, increased content of fibre (from 24 g AOAC to 30 g AOAC) and a decrease in energy component (from 8950 kJ to 8400 kJ). All calculations were carried out by the first author and then later independently checked by another researcher (BA).

We used the Nova framework to classify foods as UPF or not, using assignments from previous work.^20^ In this, two researchers independently classified foods into the four Nova groups. To assign the Nova group, NDNS foods were coded where possible based on their NDNS main food group (n=56). If there was uncertainty about whether all foods in a main food group would have the same Nova category, foods were categorised where possible by food subgroups (n=136). In cases of further uncertainty, individual food items (n=4555) were classified (eg, composite dishes which include individual foods from more than one main or subgroup). Any disagreement between the researchers on the classifications was resolved through discussion. This process achieved a high degree of inter-rater reliability—97% in the first and 99.8% in the second round after discussion.^20^ Food supplements (ie, vitamins and minerals) are not classified by the Nova system and we did not include them or alcoholic beverages. Home-prepared food which was made with unprocessed or minimally processed ingredients was classified as unprocessed or minimally processed food. If these foods contained breadcrumbs, which were purchased or retailed or frozen, or with margarine; or pastry which was purchased or retailed or frozen, or with margarine, they were classified as ultra-processed. Given the high level of processing, toddler food was classified as ultra-processed.^21^

### Data analyses

Reflecting our aims, our analyses were descriptive. First, we conducted a food-level analysis. This used all reported foods consumed by NDNS participants in 2008/2009–2018/2019, with foods reported multiple times being included multiple times, which effectively weights the findings for consumption. All foods, including toddler foods, were included in the data analyses, but we excluded alcohol-containing products and dietary supplements. We described the percentage of foods that were UPF, HFSS, both or neither. We present this first as a food-level analysis including all foods and then, separately, the relative contribution of UPFs and HFSS to total food energy (kcal) and total food weight (g) intake. We then present examples of food groups making the greatest contributions to each category based on all foods, total food energy (kcal) and total weight (g). We include an analysis based on total food energy to reflect that different foods are consumed in different portion sizes and have different energy densities and so make different relative contributions to the diet. This enables comparisons with other studies.^15^ We also include a metric based on total food weight to reflect that some UPFs make little contribution to energy (eg, artificially sweetened beverages), and list the NDNS main food groups making the largest contributions to each of these four categories (UPF, HFSS, both and neither).

Second, we conducted person-level analyses. These replicate the food level analyses but by including population survey weights, we additionally account for non-selection and non-response bias. All summaries include a two-sided CI for the weighted mean of data. Since current literature suggests that UPF/HFSS consumption might differ for demographic groups, we report the results across different age groups (1.5–3; 4–10; 11–18; 19–64 and 65+) and separately for males and females.

As a robustness check, we replicated the food-level analyses of foods consumed for males and females separately. The study protocol is available at https://osf.io/ydp2n, the code and the libraries for calculating HFSS scores and the analyses are available at https://github.com/VKesaite/HFSS-and-UPF. All calculations were carried out in Python V.3.11.4. With the exception of some additional subgroup analyses results (ie, addition of results across different age groups and separately for males and females), there were no substantive deviations from the protocol. The reporting of this study was conducted using the Strengthening the Reporting in Epidemiology guidelines.

## Results

A total of 15 655 individuals (7207 males and 8448 females) were included in the analysis. They reported consuming 1 730 158 foods, representing 4555 unique food names. For subgroup analyses, we included 717 males and 658 females aged 1.5–3; 1571 males and 1440 females aged 4–10; 1619 males and 1651 females aged 11–18; 2519 males and 3617 females aged 19–64; and 781 males and 1082 females aged 65+. We report findings based on the 2004/2005 NPM here and on the 2018 NPM in supplemental material.

### Food-level analysis

**Figure 1:**
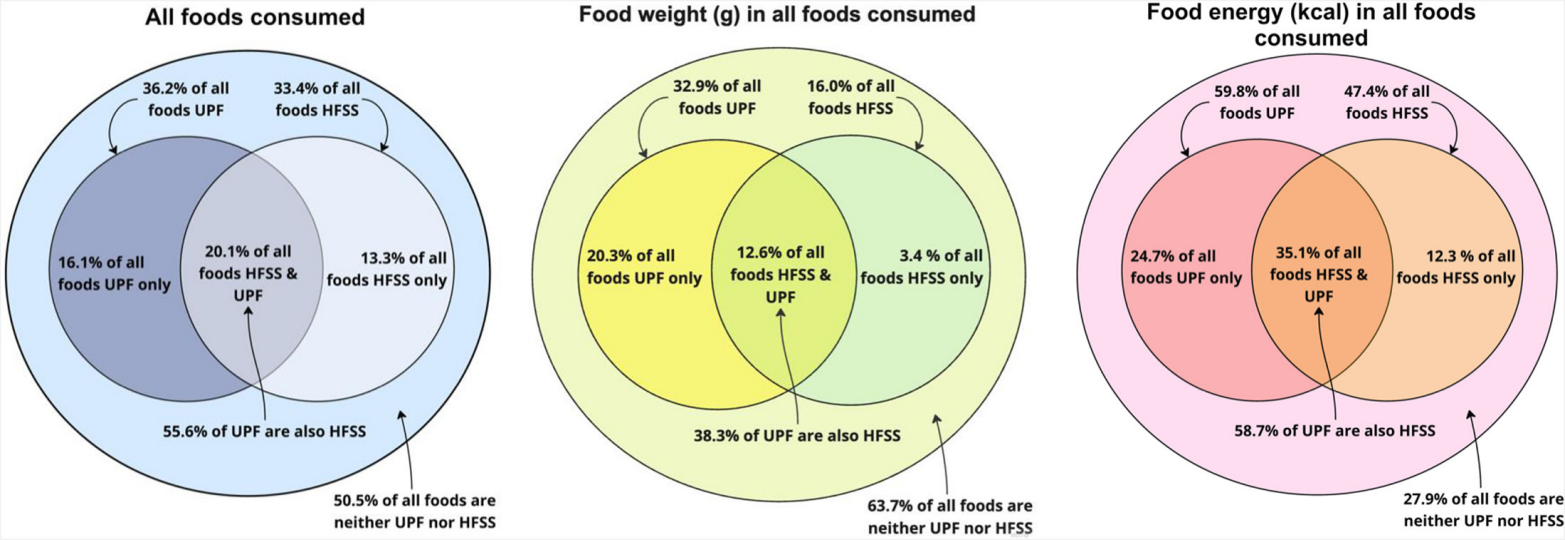
The percentage of (a) all foods, (b) food energy in kcal and (c) food weight in grams consumed that was derived from foods that are HFSS, UPF, both or neither; UK National Diet and Nutrition Survey 2008/2009–2018. HFSS, high in fat, salt or sugar (using the 2004/2005 nutrient profiling model); UPF, ultra-processed food.

Figure 1 shows Venn diagrams describing the proportions of (**a**) foods, (**b**) food energy and (**c**) food weight that were HFSS (right inner circle), UPF (left inner circle), both (inner circle overlap) and neither (outer circle). The proportion of UPFs that are HFSS is also shown. In total, 33.4% of foods, 47.4% of energy and 16.0% of food weight were HFSS, while 36.2% of food, 59.8% of energy and 32.9% of food weight were UPF. We found that 55.6% of UPFs, 58.7% of energy from UPFs and 38.3% of UPF weight were also HFSS. Robustness checks in males and females separately showed similar findings (online supplemental table S1). Analysis using the 2018 version of the NPM led to broadly similar results, but in general, a slightly smaller proportion of UPF was categorised as HFSS compared with the 2004/2005 NPM (online supplemental table S1).

Table 1 lists the 10 main food groups making the greatest contributions to the categories HFSS only, UPF only, both and neither for all foods. Tables2  3 show similar data for food energy and food weight, respectively. The most common main food groups contributing to foods and food energy that were HFSS but not UPF reflected products high in sugars (ie, ‘sugars, preserves and sweet spread’) and dairy products (or replacements) such as cheese, butter, oil, butter and oil replacements and whole milk. When analysed by food weight, the most common food groups contributing to the HFSS only category were dairy products such as whole milk, cheese, butter and cream products and also sugars (ie, ‘sugars, preserves and sweet spread’), bacon and ham products, and starchy products (ie, ‘pasta, rice and other cereals’).

**Table 1:** The 10 main food groups (% contribution) making the greatest contributions to the categories: high in fat, salt or sugar only, ultra-processed food only, both and neither at the food level for all foods; UK NDNS 2008/2009–2018/2019

All foods consumed			
**HFSS only**	**UPF only**	**HFSS and UPF**	**Neither HFSS nor UPF**
Sugars, preserves and sweet spread (26.6%)	Soft drinks, low calorie (21.3%)	Miscellaneous* (14.0%)	Tea, coffee and water (31.0%)
Miscellaneous* (13.1%)	White bread (17.5%)	Biscuits (10.1%)	Vegetables not raw (13.0%)
Cheese (10.9%)	Miscellaneous* (5.9%)	Reduced fat spread (9.6%)	Semiskimmed milk (12.3%)
Butter (10.4%)	Chips, fried and roast potatoes, potato products (5.8%)	Soft drinks, not low calorie (8.2%)	Fruit† (9.8%)
Other margarine, fats and oils¶ (9.2%)	High fibre breakfast cereals (5.5%)	Crisps and savoury snacks (6.1%)	Salad and other raw vegetables (7.9%)
Whole milk (7.9%)	Brown, granary and wheatgerm bread (5.5%)	Chocolate confectionery (6.1%)	Pasta, rice and other cereals (4.3%)
Bacon and ham (4.1%)	Yoghurt, fromage frais and dairy desserts (5.4%)	Buns, cakes, pastries and fruit pies (5.4%)	Miscellaneous* (3.4%)
Polyunsaturated fatty acid margarine and oils (3.8%)	Wholemeal bread (5.3%)	Bacon and ham (3.6%)	Other potatoes, potato salads and dishes** (2.6%)
Nuts and seeds‡ (2.9%)	Vegetables not raw (4.0%)	Other breakfast cereals§ (3.5%)	Fruit juice (2.6%)
Other milk and cream† (2.7%)	Artificial sweeteners (3.5%)	Sugars, preserves and sweet spreads (3.5%)	Chicken and turkey dishes (2.3%)

main food groups are those used in the National Diet and Nutrition Survey.

* Miscellaneous includes, for example, mayonnaise, sour cream dip, soups.

† Fruit includes, for example, raw fruit, fruit stewed with and without sugar, fruit canned in fruit and sugar syrup.

‡ Nuts and seeds includes sesame seeds, unsalted, salted and roasted peanuts; †Other milk and cream includes, for example, plant-based milk alternatives, coffee creamer, crème fraiche.

§ Other breakfast cereals includes, for example, special flakes, rice crispies.

¶ Other margarine, fats and oils includes, for example, vegetable suet, palm oil, chicken fat.

** Other potatoes, potato salads and dishes includes potato curry, mash potato with butter and cream.

HFSS, high in fat, salt or sugar (using the 2004/2005 nutrient profiling model); UPF, ultra-processed food.

**Table 2:** The 10 main food groups (% contribution) making the greatest contributions to the categories: high in fat, salt or sugar only, ultra-processed food only, both and neither at the food level for food energy (kcal); UK NDNS 2008/2009–2018/2019

Food energy (kcal) in all foods consumed			
**HFSS only**	**UPF only**	**HFSS and UPF**	**Neither HFSS nor UPF**
Cheese (22.2%)	White bread (27.8%)	Biscuits (11.2%)	Pasta, rice and other cereals (16.2%)
Butter (14.6%)	Chips fried and roast potatoes and potato products (12.7%)	Buns, cakes, pastries and fruit pies (10.0%)	Fruit* (11.9%)
Sugars, preserves and sweet spreads (12.9%)	Brown granary and wheatgerm bread (8.4%)	Chocolate confectionery (7.8%)	Semi skimmed milk (10.8%)
Whole milk (8.6%)	Wholemeal bread (7.3%)	Crisps and savoury snacks (7.2%)	Chicken and turkey dishes (9.3%)
Bacon and ham (8.2%)	High fibre breakfast cereals (6.9%)	Pasta, rice and other cereals (7.1%)	Other potatoes, potato salads and dishes† (7.7%)
Other margarine, fats and oils‡ (6.0%)	Pasta, rice and other cereals (5.5%)	Soft drinks, not low calorie (7.0%)	Vegetables not raw (7.0%)
Nuts and seeds§ (4.3%)	Vegetables not raw (4.3%)	Miscellaneous¶ (5.8%)	Beef, veal and dishes (5.5%)
Other milk and cream** (3.0%)	Yoghurt, fromage, frais and dairy desserts (3.9%)	Sausages (5.4%)	Eggs and egg dishes (4.7%)
Buns, cakes, pastries (2.2%)	Miscellaneous¶ (3.5%)	Reduced fat spread (5.1%)	Fruit juice (4.3%)
Polyunsaturated fatty acid margarine and oils (2.1%)	Coated chicken (3.3%)	Meat pies and pastries (4.1%)	Chips fried and roast potatoes, and potato products (4.1%)
Pasta, rice and other cereals (2.0%)	Other milk and cream** (1.9%)	Other breakfast cereals†† (3.8%)	Whole milk (4.0%)

Main food groups are those used in the National Diet and Nutrition Survey.

* Fruit includes, for example, raw fruit, fruit stewed with and without sugar, fruit canned in fruit and sugar syrup

† Other potatoes, potato salads and dishes includes potato curry, mash potato with butter and cream

‡ Other margarine, fats and oils includes, for example, vegetable suet, palm oil, chicken fat

§ Nuts and seeds includes sesame seeds, unsalted, salted and roasted peanuts

¶ Miscellaneous includes e.g. mayonnaise, sour cream dip, soups

** Other milk and cream includes, for example, plant-based milk alternatives, coffee creamer, crème fraiche

†† Other breakfast cereals includes e.g. special flakes, rice crispies

HFSS, high in fat, salt or sugar (using the 2004/2005 nutrient profiling model); UPF, ultra-processed food.

**Table 3:** The 10 main food groups (% contribution) making the greatest contributions to the categories: high in fat, salt or sugar only, ultra-processed food only, both and neither at the food level for food weight (g); UK NDNS 2008/2009–2018/2019

Food weight (g) in all foods consumed			
**HFSS only**	**UPF only**	**HFSS and UPF**	**Neither HFSS nor UPF**
Whole milk (34.4%)	Soft drinks, low calorie (35.4%)	Soft drinks, not low calorie (34.3%)	Tea, coffee and water (57.3%)
Cheese (15.2%)	White bread (9.9%)	Pasta, rice and other cereals (6.2%)	Semiskimmed milk (7.9%)
Sugars, preserves and sweet spreads (9.3%)	Soft drinks, not low calorie (6.5%)	Miscellaneous* (5.7%)	Fruit† (6.7%)
Bacon and ham (9.1%)	Miscellaneous* (6.1%)	Buns, cakes, pastries and fruit pies (5.6%)	Vegetables, not raw (5.6%)
Butter (5.4%)	Chips fried and roast potatoes, and potato products (5.5%)	Biscuits (5.1%)	Fruit juice (3.7%)
Other milk and cream‡ (2.4%)	Yoghurt, fromage frais and dairy desserts (4.7%)	Sausages (4.3%)	Pasta, rice, and other cereals (3.3%)
Pasta, rice and other cereals (2.0%)	Vegetables not raw (4.3%)	Yoghurt, fromage frais and dairy desserts (3.4%)	Other potatoes, potato salads and dishes§ (3.1%)
Nuts and seeds** (2.0%)	Tea, coffee and water (3.4%)	Chocolate confectionery (3.3%)	Whole milk (2.1%)
Other margarine, fats and oils†† (1.8%)	Pasta, rice and other cereals (3.4%)	Crisps and savoury snacks (3.0%)	Salad and other raw vegetables (2.0%)
White fish coated or fried (1.7%)	Brown granary and wheatgerm bread (3.1%)	Ice cream (2.9%)	Chicken and turkey dishes (1.9%)

Main food groups are those used in the National Diet and Nutrition Survey.

* Miscellaneous includes, for example, mayonnaise, sour cream dip, soups

† Fruit includes e.g. raw fruit, fruit stewed with and without sugar, fruit canned in fruit and sugar syrup

‡ Other milk and cream includes e.g. plant-based milk alternatives, coffee creamer, crème fraiche

§ Other potatoes, potato salads and dishes includes potato curry, mash potato with butter and cream

¶ Nuts and seeds includes sesame seeds, unsalted, salted and roasted peanut

** Nuts and seeds includes sesame seeds, unsalted, salted and roasted peanuts

†† Other margarine, fats and oils includes e.g. vegetable suet, palm oil, chicken fat

HFSS, high in fat, salt or sugar (using the 2004/2005 nutrient profiling model); UPF, ultra-processed food.

The most common main food groups contributing to foods in the category UPF but not HFSS often included non-nutritive sweeteners (ie, ‘soft drinks low calorie’, ‘artificial sweeteners’) or were breads including white, brown, granary and wholemeal bread. Fried and roast potatoes and potato products were also a common food group in this category. When considering food weight and food energy, the most common food groups in the UPF only category were similar to the total food category.

In the category of foods that were UPF and HFSS, the most common main groups reflected miscellaneous foods (eg, condiments and soups), bakery products (eg, biscuits and ‘buns, cakes, pastries and fruit pies’), reduced fat spread, and ‘soft drinks, not low calorie’. When considering by food energy and food weight, products high in sugar (biscuits, ‘buns, cakes, pastries and fruit pies’, chocolate confectionery), ‘crisps and savoury snacks’ and ‘pasta, rice and other cereals’ made substantial contributions to the category that was both UPF and HFSS.

The most common main food group contributing to the category of foods that were neither HFSS nor UPF was tea, coffee and water. Cooked and raw vegetables, semiskimmed milk and fruit were also prominent. Similar findings were seen for food weight. By food energy, the food groups making the largest contribution to this category included pasta, rice and other cereals, fruit, semiskimmed milk and chicken and turkey dishes.

### Person-level analysis

The results of the person-level analyses across different age groups and for males and females are summarised in tables4^7^, while aggregate level summaries are presented in online supplemental table S3. Additionally, including sampling weights led to small changes compared with the estimates in figure 1. At person level, 55.1% of UPF foods are also HFSS (c.f. 55.6% at food level), 53.8% of UPF energy is also HFSS (c.f. 58.7%) and 40.0% of UPF food weight is also HFSS (c.f. 38.3%). Subgroup analyses by age and gender suggest that among toddlers (aged 1.5–3), nearly 60% of food energy is derived from UPFs, and about 40% from HFSS. Among children (aged 4–10) food energy derived from HFSS and UPF foods is equally high—more than 60% of all energy is obtained from UPF foods and nearly half is from HFSS foods. Similarly, among youth (aged 11–18), food energy derived from UPF foods is 65.6% among males and 65.1% among females and from HFSS foods it is 49.5% among males and 49.2% among females. Among individuals aged 19–64, food energy derived from UPF foods amounts to 55% for males and 51.6% for females, while HFSS foods account for about 43.4% for males and 42.1% for females. Similar results are obtained among individuals aged 65+, food energy derived from HFSS foods is around 42.1% for males and 41.2% for females, while food energy derived from UPF foods is marginally larger than for HFSS, 51.7% for males and 49.5% for females.

**Table 4:** The percentage of per capita daily foods for males and females (aged 1.5–10), food energy in kcal, and food weight in grams consumed that was derived from foods that are HFSS, UPFs, both and neither (95% CI); UK NDNS 2008/2009–2018/2019

	Males	Females	Males	Females	Males	Females
	**All foods consumed; 95%** CI	**All foods consumed; 95%** CI	**Food energy (kcal); 95%** CI	**Food energy (kcal); 95%** CI	**Food weight (g); 95%** CI	**Food weight (g); 95%** CI
	Children aged 1.5 to 3					
Neither UPFs nor HFSS	42.4 (41.2 to 43.5)	41.0 (39.8 to 42.2)	31.1 (30.0 to 32.2)	30.2 (29.0 to 31.4)	50.8 (49.1 to 52.5)	49.8 (48.2 to 51.5)
All UPF	45.6 (44.2 to 47.0)	47.2 (45.9 to 48.6)	56.4 (55.2 to 57.6)	57.3 (56.0 to 58.5)	41.3 (39.7 to 42.9)	42.5 (40.9 to 44.1)
UPF only	23.9 (22.9 to 24.9)	24.5 (23.5 to 25.6)	26.4 (25.5 to 27.3)	26.9 (26.0 to 27.7)	30.6 (29.7 to 31.4)	31.3 (30.5 to 32.1)
All HFSS	33.8 (33.0 to 34.5)	34.5 (33.7 to 35.2)	42.5 (41.4 to 43.5)	42.9 (41.9 to 44.0)	18.6 (17.6 to 19.6)	18.8 (17.8 to 19.8)
HFSS only	12.0 (11.5 to 12.6)	11.8 (11.1 to 12.4)	12.5 (11.7 to 13.3)	12.5 (11.7 to 13.4)	7.9 (7.0 to 8.8)	7.7 (6.8 to 8.6)
HFSS and UPFs	21.7 (21.0 to 22.4)	22.7 (21.9 to 23.4)	30.0 (29.1 to 30.9)	30.4 (29.4 to 31.5)	10.7 (10.2 to 11.2)	11.1 (10.6 to 11.7)
UPF that is also HFSS	49.4 (48.3 to 50.5)	49.1 (48.0 to 50.2)	52.9 (51.7 to 54.2)	51.8 (50.5 to 53.1)	31.1 (29.8 to 32.4)	29.6 (28.3 to 30.9)
	Children aged 4 to 10					
Neither UPFs nor HFSS	39.3 (38.6 to 39.9)	41.4 (40.7 to 42.1)	26.0 (25.4 to 26.5)	27.1 (26.5 to 27.7)	50.7 (49.8 to 51.6)	53.5 (52.6 to 54.4)
All UPF	50.1 (49.3 to 50.9)	47.5 (46.7 to 48.4)	64.5 (63.8 to 65.1)	63.0 (62.3 to 63.7)	44.8 (43.9 to 45.7)	42.0 (41.1 to 43.0)
UPF only	22.1 (21.6 to 22.6)	20.6 (20.1 to 21.1)	25.6 (25.1 to 26.1)	23.9 (23.5 to 24.4)	28.4 (27.6 to 29.2)	26.1 (25.3 to 26.9)
All HFSS	38.6 (38.1 to 39.1)	38.0 (37.5 to 38.5)	48.4 (47.9 to 49.0)	49.0 (48.4 to 49.6)	20.9 (20.3 to 21.4)	20.4 (19.8 to 20.9)
HFSS only	10.7 (10.3 to 11.0)	11.0 (10.7 to 11.4)	9.5 (9.2 to 9.9)	9.9 (9.5 to 10.3)	4.5 (4.2 to 4.9)	4.4 (4.1 to 4.8)
HFSS and UPFs	27.9 (27.4 to 28.4)	26.9 (26.4 to 27.5)	38.9 (38.3 to 39.5)	39.1 (38.4 to 39.7)	16.4 (15.9 to 16.8)	15.9 (15.5 to 16.3)
UPF that is also HFSS	56.3 (55.7 to 57.0)	57.3 (56.6 to 58.0)	59.7 (59.0 to 60.4)	61.3 (60.6 to 62.0)	38.9 (38.0 to 39.7)	40.8 (39.9 to 41.7)

HFSS (using the 2004/2005 nutrient profiling model); data are weighted to correct for non-random and non-response selection of households.

HFSS, high in fat, salt or sugar; NDNS, National Diet and Nutrition Survey; UPF, ultra-processed food.

**Table 5:** The percentage of per capita daily foods for males and females (aged 11–18), food energy in kcal, and food weight in grams consumed that was derived from foods that are HFSS, UPFs, both and neither (95% CI); UK NDNS 2008/2009–2018/2019

	Males	Females	Males	Females	Males	Females
	**All foods consumed; 95%** CI	**All foods consumed; 95%** CI	**Food energy (kcal); 95%** CI	**Food energy (kcal); 95%** CI	**Food weight (g); 95%** CI	**Food weight (g); 95%** CI
Neither UPFs nor HFSS	39.7 (39.0 to 40.3)	41.5 (40.8 to 42.1)	24.6 (24.1 to 25.2)	25.1 (24.5 to 25.7)	50.3 (49.4 to 51.3)	53.1 (52.1 to 54.1)
All UPF	48.3 (47.5 to 49.1)	46.9 (46.1 to 47.7)	65.6 (65.0 to 66.3)	65.1 (64.4 to 65.8)	46.2 (45.3 to 47.2)	43.6 (42.7 to 44.6)
UPF only	19.9 (19.5 to 20.4)	19.5 (19.0 to 20.0)	25.8 (25.3 to 26.3)	25.6 (25.1 to 26.2)	24.9 (24.2 to 25.6)	24.2 (23.5 to 25.0)
All HFSS	40.4 (39.9 to 40.9)	39.0 (38.5 to 39.5)	49.5 (48.9 to 50.2)	49.2 (48.6 to 49.8)	24.8 (24.2 to 25.5)	22.7 (22.0 to 23.3)
HFSS only	12.0 (11.7 to 12.4)	11.7 (11.3 to 12.0)	9.7 (9.4 to 10.1)	9.8 (9.4 to 10.1)	3.4 (3.2 to 3.7)	3.3 (3.0 to 3.5)
HFSS and UPFs	28.4 (27.8 to 28.9)	27.4 (26.8 to 27.9)	39.8 (39.1 to 40.5)	39.5 (38.8 to 40.1)	21.4 (20.8 to 22.0)	19.4 (18.8 to 20.0)
UPF that is also HFSS	58.8 (58.2 to 59.5)	58.6 (57.9 to 59.3)	60.0 (59.2 to 60.7)	60.1 (59.4 to 60.9)	46.2 (45.3 to 47.2)	45.8 (44.8 to 46.7)

HFSS (using the 2004/2005 nutrient profiling model); data are weighted to correct for non-random and non-response selection of households.

HFSS, high in fat, salt or sugar; NDNS, National Diet and Nutrition Survey; UPF, ultra-processed food.

**Table 6:** The percentage of per capita daily foods for males and females (aged 19–64), food energy in kcal, and food weight in grams consumed that was derived from foods that are HFSS, UPFs, both and neither (95% CI); UK NDNS 2008/2009 to 2018/2019

	Males	Females	Males	Females	Males	Females
	**All foods consumed; 95%** CI	**All foods consumed; 95%** CI	**Food energy (kcal); 95%** CI	**Food energy (kcal); 95%** CI	**Food weight (g); 95%** CI	**Food weight (g); 95%** CI
Neither UPFs nor HFSS	53.9 (53.6 to 54.2)	57.1 (56.8 to 57.4)	31.3 (30.8 to 31.8)	34.5 (34.0 to 35.0)	67.0 (66.4 to 67.6)	71.6 (71.0 to 72.2)
All UPF	31.6 (31.1 to 32.1)	29.2 (28.8 to 29.7)	55.0 (54.4 to 55.6)	51.6 (51.0 to 52.1)	29.6 (29.2 to 30.0)	25.5 (25.1 to 25.9)
UPF only	13.9 (13.7 to 14.1)	13.3 (13.1 to 13.5)	25.3 (25.0 to 25.7)	23.4 (23.0 to 23.8)	17.5 (17.1 to 18.0)	16.0 (15.6 to 16.5)
All HFSS	32.2 (31.8 to 32.6)	29.6 (29.2 to 30.0)	43.4 (42.9 to 43.8)	42.1 (41.6 to 42.6)	15.5 (15.1 to 15.9)	12.4 (12.0 to 12.7)
HFSS only	14.5 (14.3 to 14.7)	13.7 (13.5 to 13.9)	13.7 (13.4 to 14.0)	13.9 (13.6 to 14.2)	3.4 (3.3 to 3.5)	2.9 (2.8 to 3.0)
HFSS and UPFs	17.7 (17.3 to 18.0)	15.9 (15.6 to 16.2)	29.6 (29.1 to 30.1)	28.2 (27.7 to 28.7)	12.1 (11.8 to 12.3)	9.5 (9.3 to 9.7)
UPF that is also HFSS	55.2 (54.9 to 55.7)	55.1 (54.5 to 55.4)	52.3 (51.6 to 52.9)	53.9 (53.2 to 54.5)	40.9 (40.2 to 41.6)	39.4 (38.7 to 40.1)

HFSS (using the 2004/2005 nutrient profiling model); data are weighted to correct for non-random and non-response selection of households.

HFSS, high in fat, salt or sugar; NDNS, National Diet and Nutrition Survey; UPF, ultra-processed food.

**Table 7:** The percentage of per capita daily foods for males and females (aged 65+), food energy in kcal and food weight in grams consumed that was derived from foods that are HFSS, UPFs, both and neither (95% CI); UK NDNS 2008/2009 to 2018/2019

	Males	Females	Males	Females	Males	Females
	**All foods consumed; 95%** CI	**All foods consumed; 95%** CI	**Food energy (kcal); 95%** CI	**Food energy (kcal); 95%** CI	**Food weight (g); 95%** CI	**Food weight (g); 95%** CI
Neither UPFs nor HFSS	56.8 (56.1 to 57.6)	60.1 (59.4 to 60.7)	32.5 (31.6 to 33.3)	34.9 (34.2 to 35.7)	74.6 (73.8 to 75.3)	77.8 (77.2 to 78.5)
All UPF	28.3 (27.6 to 29.0)	26.7 (26.1 to 27.3)	51.7 (50.8 to 52.6)	49.5 (48.6 to 50.3)	21.7 (21.0 to 22.4)	19.1 (18.5 to 19.7)
UPF only	13.2 (12.8 to 13.7)	12.4 (12.0 to 12.8)	25.4 (24.8 to 26.1)	23.8 (23.2 to 24.5)	13.8 (13.2 to 14.4)	12.4 (11.9 to 12.9)
All HFSS	29.9 (29.2 to 30.6)	27.5 (27.0 to 28.1)	42.1 (41.2 to 43.0)	41.2 (40.5 to 42.0)	11.6 (11.2 to 12.1)	9.7 (9.4 to 10.1)
HFSS only	14.9 (14.3 to 15.5)	13.2 (12.7 to 13.7)	15.8 (15.2 to 16.5)	15.6 (15.0 to 16.2)	3.7 (3.5 to 4.0)	3.1 (2.9 to 3.3)
HFSS and UPFs	15.0 (14.6 to 15.5)	14.3 (13.9 to 14.7)	26.3 (25.5 to 27.1)	25.6 (24.9 to 26.4)	7.9 (7.5 to 8.3)	6.7 (6.4 to 6.9)
UPF that is also HFSS	53.0 (51.9 to 54.1)	54.0 (53.0 to 55.0)	49.5 (48.4 to 50.7)	51.1 (50.0 to 52.2)	38.0 (36.8 to 39.3)	38.4 (37.3 to 39.5)

HFSS=(using the 2004/05 nutrient profiling model); data are weighted to correct for non-random and non-response selection of households.

HFSS, high in fat, salt or sugar; NDNS, National Diet and Nutrition Survey; UPF, ultra-processed food.

Across all age groups considered, males had a slightly higher consumption of UPF/HFSS compared with females. Similarly, food energy derived from UPF and HFSS increased slightly with age and was highest in 11–18 years but then decreased slightly in adulthood (19+ years).

Analysis using the 2018 version of the NPM led to broadly similar results but, overall, a slightly smaller proportion of UPF was categorised as HFSS compared with the 2004/2005 NPM (online supplemental table S2). Since policy-makers are currently using the NPM 2004/2005, we report most of the results based on the NPM 2004/2005.

## Discussion

### Summary of main findings

This is the first study to assess the extent to which foods consumed in the UK are HFSS, UPFs, both or neither. Using 11 years of data from NDNS, we found that around one-third of all foods consumed (33.4%) were HFSS, one-third (36.2%) were UPF, one-fifth were both (20.1%) and half (50.5%) neither. We found that 55.6% of UPFs, 58.7% of food energy and 38.3% of food weight consumed were also HFSS. This means that policies focused on HFSS reduction also target between one-third and just over a half of UPFs.

When considering all foods consumed, common foods that were UPF but not HFSS (and so would not be included in HFSS-focused policies) included low-calorie soft drinks, breads and high-fibre breakfast cereals. Food groups making substantial contributions to all foods consumed in the category HFSS and UPF were miscellaneous (eg, condiments and soups), biscuits, reduced fat spread and ‘soft drinks, not low calorie’.

Consistent with previous studies,^22^ we found that HFSS and UPF consumption was marginally higher among men than women and among younger age groups (≤18 years) than older ages (≥19 years).

### Strengths and limitations

To the best of our knowledge, this is the first study to classify all foods consumed in the UK as both HFSS and UPF and compare these classifications. We used food-level dietary data, with 3 or 4 food diary days per person from the NDNS. Food diaries reduce the recall bias common in food frequency questionnaires and 24-hour recalls. We used three metrics of food consumption—total number of foods, food energy and food weight. Analysing food energy recognises that different foods are consumed in different portion sizes while assessing food weight identifies, in particular, foods that have a high weight (eg, drinks) but low energy ingredients (eg, non-nutritive sweeteners).

While the accuracy of food classification using the Nova system has been criticised as poorly replicable,^23^ we and others have achieved a high degree of inter-rater reliability.^20 24^ Furthermore, by including all foods reported by NDNS participants (excluding alcohol and supplements), we effectively weighted by frequency of consumption. The additional inclusion of sampling weights in individual-level analyses did not change the overall pattern of findings, indicating that our findings are likely to be generalisable to the UK population.

Despite their strengths, food diaries are self-reported, leading to the potential for measurement error. Further, while we included 11 years of data, the most recent was from 2019. Given the increasing number of new foods commercially available,^25^ we may not have captured recent changes in food consumption. Moreover, combining 11 years of data might have masked important changes over time but ensured a large sample size. NDNS did not collect data from new participants in 2020 and subsequently changed dietary data collection method, which meant that newer comparable data are not available.

There are also some potential limitations with the nutritional measures used in this study. The Nova classification has been criticised for its lack of consideration of nutritional content and quality. With some foods, ultra-processing might yield nutritional and health benefits such as micronutrient fortification, increased affordability, extended shelf life and reduced waste.^26^ A recent study indicated that while consumption of some UPFs, such as artificially and sugar-sweetened beverages, was associated with an increased risk of cancer and cardiometabolic diseases, no evidence of an association was found for others such as UPF bread and cereals.^1^ Our second measure of nutritional quality, HFSS has also been criticised as oversimplified.^27^ Another limitation is the lack of information on food branding in NDNS. While we applied UPF and HFSS classifications to the generic foods and their nutrient content listed in NDNS (eg, white bread), it is possible that not all forms of these foods have the same UPF or nutrient content and hence HFSS status. Moreover, while the NPM was designed around the guideline daily amounts (GDAs) for children between the ages 11 and 16, it has been found to be applicable to the dietary needs of younger children aged 4–16 as well as adults.^28^ We are not aware of any work exploring applicability to the GDAs of younger children.

### Interpretation and implications

We observed that, at best, 58.7% of UPFs consumed were identified as HFSS. This is a lower degree of overlap than a recent US study which found that 75.4% of UPFs were also HFSS.^15^ These differences may reflect the different food supply, purchase and consumption patterns seen in the UK and USA. It may also reflect methodological differences. For instance, Popkin *et al*^15^ focused on the presence of food additives when defining UPF, while in this study, we used a wider range of ingredient-level information.

We find that extending the definition of HFSS to include non-nutritive sweeteners would increase the proportion of UPF captured by HFSS to just over two-thirds. Similar findings were reported by Popkin *et al*.^15^ While some recent UK public health nutrition policies have increased the number of products available that contain non-nutritive sweeteners,^29^ the WHO has recently noted that these may not be useful for weight loss.^30^ There may also be other ways that the HFSS algorithm could be adapted to capture more UPFs such as by incorporating components such as non-nutritive sweeteners, colours/flavours or other additives.^15^

When analysing by food energy, rather than total number of foods, the ‘other milk and cream’ food group was among the top 10 food groups contributing to the category of UPF but not HFSS. This includes plant-based milk alternatives. Some plant-based alternatives can be healthier than equivalent meat and dairy products,^31^ and some plant-based alternatives are UPF. While there is uncertainty about the environmental impacts of UPF as a category,^32^ reducing consumption of animal-based products at a population level will be essential to achieve net-zero. UPF plant-based alternatives could be a stepping stone to less processed plant-based alternatives.^33^ This highlights the trade-offs common in individual dietary choices as well as policy-making and why simply dichotomising foods into those that should be consumed or avoided is challenging, however, that dichotomisation is achieved. Nevertheless, food profiling methods that consider both human and planetary health would allow for a more holistic approach, and regulating foods on sustainability grounds might have health cobenefits.^34^

Much policy in the UK has focused on food reformulation, whereby manufacturers are incentivised to change the composition of foods to ensure they are not HFSS.^35^ Other policies have focused specifically on reducing sugar and calorie content.^36^ While there is evidence that some of these policies have prompted reformulation (eg, removal of sugar from soft drinks)^37^ and have been associated with overall reduction in free sugar consumption^38^ and health benefits,^39^ reformulated foods which are no longer HFSS often remain UPF (eg, artificially sweetened soft drinks). An alternative approach using policies that incentivise deformulation^26^ (ie, removing components and additives commonly used in UPFs, such as non-nutritive sweeteners and emulsifiers) could help reduce production and consumption of UPF. Any such policies should be sensitive to impacts on price and health inequalities. Accompanying such policy approaches with those that incentivise whole food consumption could offer important synergies. A combination of policies that incentivise healthy eating should be used, including taxation, subsidies and incentives or nudges that promote healthy eating.^40^ Furthermore, better understanding of ways to improve the supply and availability of healthy food is needed.^40^

Our results reinforce the well-established finding that current UK diets are suboptimal for health. We found that 54.9% of per capita daily energy was derived from UPFs and 43.7% from HFSS foods. This is comparable to previous findings. For instance, a previous study in the UK based on NDNS data from 2008 to 14 found that 56.8% of energy was derived from UPFs.^41^ Similarly, in Canada, 47.7% of energy was derived from UPFs,^42^ and in the USA, 57.5% was derived from UPFs.^43^ Studies from other countries find a smaller proportion of energy attributable to UPFs. For example, a study from Italy found that 17.3% of energy was derived from UPFs.^44^ Current UK dietary guidance advises citizens to eat HFSS foods ‘less often and in small amounts’.^45^

## Conclusions

In this analysis of 11 years of data from the UK NDNS, we found that the NPM, used to identify foods that are HFSS for regulatory purposes identifies, at best, 58.7% of UPFs. If UK policy-makers decide that regulation of UPFs is necessary, additional action will be required to extend current policy. This could involve extending the current NPM to include ingredients, such as non-nutritive sweeteners and emulsifiers, that are common in foods that are UPF but not currently identified as HFSS. Further work is required to confirm that UPF consumption is causally associated with health harms and to determine the environmental impacts of UPF.

## Supplementary material

10.1136/bmjnph-2024-001035 online supplemental file 1

## Data Availability

Data are available in a public, open access repository.
