# Supplementary material for: Overlap between ultra-processed food and food that is high in fat, salt or sugar: analysis of 11 annual waves of the UK National Diet and Nutrition Survey 2008/2009–2018/2019
Source: BMJ Nutr Prev Health. 2025 Mar 28;8(1):e001035. doi: 10.1136/bmjnph-2024-001035 (PMC12322569; doi:10.1136/bmjnph-2024-001035)
Supplement: online supplemental file 1 [file bmjnph-8-1-s001.pdf]

Table S1. The percentage of all foods, food energy in kcal, and food weight in grams consumed that was derived from foods that are high in fat, salt or sugar, ultra-processed foods, both and neither; UK National Diet and Nutrition Survey 2008/9-2018/19

	All foods consumed; %			Food energy (kcal); %			Food weight (g); %		
	All participants	Male participants	Female participants	All participants	Male participants	Female participants	All participants	Male participants	Female participants
**Neither UPFs nor HFSS (NPM 2004/5)**	50.5	48.2	52.4	27.9	26.8	29.0	63.7	60.7	66.4
**Neither UPFs nor HFSS (NPM 2018)**	51.7	49.4	53.6	28.9	27.9	30.0	62.8	59.8	65.5
**All UPF**	36.2	38.2	34.6	59.8	61.0	58.6	32.9	35.6	30.5
**UPF only (NPM 2004/5)**	16.1	16.9	15.3	24.7	25.3	24.1	20.3	21.5	19.2
**UPF only (NPM 2018)**	16.5	17.3	15.8	27.3	28.1	26.6	19.5	20.7	18.4
**All HFSS (NPM 2004/5)**	33.4	34.8	32.2	47.4	47.9	46.9	16.0	17.8	14.4
**All HFSS (NPM 2018**)	31.8	33.2	30.6	43.7	44.0	43.5	17.7	19.5	16.1
**HFSS only (NPM 2004/5**)	13.3	13.6	13.0	12.3	12.2	12.3	3.4	3.8	3.5
**HFSS only (NPM 2018)**	12.1	12.4	11.8	11.3	11.1	11.4	4.3	4.6	4.6
**HFSS and UPF (NPM 2004/05)**	20.1	21.2	19.2	35.1	35.7	34.5	12.6	14.1	11.3
**HFSS and UPF (NPM 2018)**	19.7	20.8	18.8	32.5	32.9	32.0	13.4	14.8	12.1
**UPF that is also HFSS (NPM 2004/5)**	55.6	55.6	55.6	58.7	58.5	58.9	38.3	39.5	37.2
**UPF that is also HFSS (NPM 2018)**	54.4	54.6	54.2	54.3	53.9	54.7	40.7	35.7	39.6

Note: HFSS = high in fat, salt or sugar; UPF = ultra-processed food; data are weighted to correct for non-random non-response of households; NPM = nutrient profiling model

Table S2. The percentage of per capita daily foods, food energy in kcal, and food weight in grams consumed that was derived from foods that are high in fat, salt or sugar, ultra-processed foods, both and neither; UK National Diet and Nutrition Survey 2008/9-2018/19

	All foods consumed; % (SD)	Food energy (kcal); % (SD)	Food weight (g); % (SD)
**Neither UPFs nor HFSS (NPM 2004/5)**	52.9 (14.4)	31.7 (13.6)	66.8 (18.6)
**Neither UPFs nor HFSS (NPM 2018)**	54.1 (14.6)	32.7 (13.9)	66.0 (18.8)
**All UPF**	33.6 (15.6)	54.9 (15.7)	29.7 (18.3)
**UPF only (NPM 2004/5)**	15.0 (8.9)	24.6 (10.5)	18.2 (13.4)
**UPF only (NPM 2018)**	15.8 (8.7)	26.8 (11.3)	17.8 (12.8)
**All HFSS (NPM 2004/5)**	32.0 (10.6)	43.7 (13.2)	15.0 (10.9)
**All HFSS (NPM 2018**)	30.0 (10.3)	40.5 (12.7)	16.2 (11.5)
**HFSS only (NPM 2004/5**)	13.5 (8.1)	13.4 (8.7)	3.5 (4.3)
**HFSS only (NPM 2018)**	12.3 (6.9)	12.4 (8.0)	4.3 (4.7)
**HFSS and UPF (NPM 2004/05)**	18.5 (10.0)	30.3 (13.8)	11.5 (10.0)
**HFSS and UPF (NPM 2018)**	17.7 (9.9)	28.1 (12.9)	11.9 (10.3)
**UPF that is also HFSS (NPM 2004/5)**	55.1 (15.5)	53.9 (17.3)	40.0 (19.0)
**UPF that is also HFSS (NPM 2018)**	52.3 (15.5)	50.7 (17.3)	40.2 (19.0)

Note. HFSS = high in fat, salt or sugar (using the 2018 Nutrient Profiling Model); UPF = ultra-processed food; data are weighted to correct for non-random non-response of households.

Table S3. The percentage of per capita daily foods, food energy in kcal, and food weight in grams consumed that was derived from foods that are high in fat, salt or sugar, ultra-processed foods, both and neither [95% CI]; UK NDNS 2008/9 to 2018/19, including all age groups

	All foods consumed; 95% CI	Food energy (kcal); 95% CI	Food weight (g); 95% CI
**Neither UPFs nor HFSS**	52.9 [52.7 to 53.2]	31.7 [31.5 to 31.9]	66.8 [66.6 to 67.1]
**All UPF**	33.6 [33.3 to 33.8]	54.9 [54.7 to 55.2]	29.7 [29.4 to 30.0]
**UPF only**	15.0 [14.9 to 15.2]	24.6 [24.5 to 24.8]	18.2 [18.0 to 18.4]
**All HFSS**	32.0 [31.9 to 32.2]	43.7 [43.6 to 43.9]	15.0 [14.8 to 15.1]
**HFSS only**	13.5 [13.4 to 13.6]	13.4 [13.2 to 13.5]	3.5 [3.4 to 3.5]
**HFSS and UPFs**	18.5 [18.4 to 18.7]	30.3 [30.1 to 30.5]	11.5 [11.4 to 11.7]
**UPF that is also HFSS**	55.1 [54.9 to 55.4]	53.9 [53.6 to 54.1]	40.0 [39.7 to 40.3]

Note: HFSS = high in fat, salt or sugar (using the 2004/05 Nutrient Profiling Model); UPF = ultra-processed food; data are weighted to correct for non-random and non-response selection of households.
